# Bombardier Enables Delivery of Short-Form Bomanins in the *Drosophila* Toll Response

**DOI:** 10.3389/fimmu.2019.03040

**Published:** 2020-01-10

**Authors:** Samuel J. H. Lin, Amit Fulzele, Lianne B. Cohen, Eric J. Bennett, Steven A. Wasserman

**Affiliations:** Section of Cell and Developmental Biology, Division of Biological Sciences, University of California, San Diego, San Diego, CA, United States

**Keywords:** *Drosophila melanogaster*, immunity, Toll, Bomanins, humoral

## Abstract

Toll mediates a robust and effective innate immune response across vertebrates and invertebrates. In *Drosophila melanogaster*, activation of Toll by systemic infection drives the accumulation of a rich repertoire of immune effectors in hemolymph, including the recently characterized Bomanins, as well as the classical antimicrobial peptides (AMPs). Here we report the functional characterization of a Toll-induced hemolymph protein encoded by the *bombardier* (*CG18067*) gene. Using the CRISPR/Cas9 system to generate a precise deletion of the *bombardier* transcriptional unit, we found that Bombardier is required for Toll-mediated defense against fungi and Gram-positive bacteria. Assaying cell-free hemolymph, we found that the Bomanin-dependent candidacidal activity is also dependent on Bombardier, but is independent of the antifungal AMPs Drosomycin and Metchnikowin. Using mass spectrometry, we demonstrated that deletion of *bombardier* results in the specific absence of short-form Bomanins from hemolymph. In addition, flies lacking Bombardier exhibited a defect in pathogen tolerance that we trace to an aberrant condition triggered by Toll activation. These results lead us to a model in which the presence of Bombardier in wild-type flies enables the proper folding, secretion, or intermolecular associations of short-form Bomanins, and the absence of Bombardier disrupts one or more of these steps, resulting in defects in both immune resistance and tolerance.

## Introduction

Innate immune pathways are found in plants, fungi, and animals and provide a rapid defense against a broad range of pathogens (1–3). In the fruit fly *Drosophila melanogaster*, the two major innate immune pathways are Toll and Imd (4–6). The Toll pathway is activated by Gram-positive bacteria with Lys-type peptidoglycan and by fungi, and is required for defense against these microbes (7–10). Conversely, the Imd pathway is activated by and plays a major role in survival against Gram-negative bacteria and Gram-positive bacteria with DAP-type peptidoglycan (11, 12). These pathways, which are both mediated by NF-κB transcription factors, are broadly conserved as initiators of innate immune responses. Activation of either pathway induces robust production of an array of immune molecules, including antimicrobial peptides (AMPs) (13–17).

AMPs are found in all kingdoms of life (18–22). These peptides have long been thought to play the principal effector role in innate immune defense due to their demonstrated *in vitro* antimicrobial activity and their marked upregulation after infection. However, recent research in *D. melanogaster* suggests that AMPs play a major role in Imd-mediated defense, but a relatively minor role in Toll-mediated immunity (23).

In contrast to the AMPs, the *Drosophila*-specific Bomanin peptides (Boms), which are highly induced after infection, are indispensable for resistance against pathogens controlled by the Toll pathway (24). *Bom*^Δ55*C*^ flies, which lack 10 of the 12 *Bom* genes, succumb to fungal and Gram-positive bacterial infections at rates indistinguishable from Toll-deficient flies (23, 24), suggesting that Boms rather than AMPs are the primary Toll effectors.

Bom peptides, like AMPs, are secreted from the fat body, the *Drosophila* immune organ, into the hemolymph, the *Drosophila* circulatory fluid. The family is comprised of three groups. The short-form peptides are 16–17 residues long and contain only the Bom motif. The tailed forms contain the Bom motif followed by a C-terminal tail. Finally, the bicipital forms consist of two Bom motifs connected by a linker region (24). *Bom*^Δ55*C*^ flies lack all six of the short-form Boms, two of the three tailed Boms, and two of the three bicipital Boms. High-level expression of short-form Boms is sufficient to rescue the sensitivity of *Bom*^Δ55*C*^ flies to *C. glabrata* infection (25). Furthermore, the absence of Toll-induced candidacidal activity in *Bom*^Δ55*C*^ hemolymph can be rescued by high-level expression of a short-form Bom (25). However, no *in vitro* antimicrobial activity has been observed with Bom peptides alone (25), suggesting that the Bomanins act in coordination with additional humoral effectors.

In this study, we demonstrate an essential role in Toll-mediated humoral defense for a previously uncharacterized hemolymph protein, Bombardier (one that deploys Boms).

## Materials and Methods

### CRISPR/Cas9 Deletion of *bombardier* Locus

The *bombardier* gene (CG18067) was deleted using CRISPR/Cas9 technology according to established protocols (26). Briefly, a pair of gRNAs designed to delete the region 2R: 20,534,248–20,536,154 were cloned into pU6-BbsI-chiRNA (Addgene plasmid #45946). Homology arms (1,017 bp left and 1,022 bp right) were cloned into pDsRed-attP (Addgene plasmid #51019). The plasmid pBS-Hsp70-Cas9 (Addgene plasmid #46294) was used as the Cas9 source. Constructs were injected into *w*^1118^ embryos. F1 progeny were screened for DsRed eyes and homozygous lines were established. See Supplemental Table 1 for gRNA and homology arm primer sequences.

### Toll Activation, *Drosophila* Infection, and Survival Analysis

Flies were raised at 25°C on cornmeal molasses agar media^1^. The *w*^1118^ strain was used as the wild type. Microbial isolates, culture conditions, and conditions for infection for *Enterococcus faecalis, Enterobacter cloacae, Fusarium oxysporum*, and *Candida glabrata* were as described previously (24), except that *C. glabrata* was concentrated to OD_600_ = 100. Flies were incubated at 25°C after live bacterial infection and at 29°C after fungal infection. For heat-killed challenge, bacterial cultures were autoclaved and resuspended in 20% glycerol to OD_600_ = 10 for *E. faecalis* and OD_600_ = 300 for *M. luteus*. For both survival assays and hemolymph preparation, flies challenged with heat-killed bacteria were incubated at 29°C.

### Hemolymph Antimicrobial Assays

Candidacidal activity of hemolymph was assayed as described previously (25), except that hemolymph was prepared from groups of 30 flies and all activity assays were carried out for 1 h at room temperature. The number of colonies representing zero percent killing was set as the value obtained by assaying uninduced *w*^1118^ hemolymph.

### MALDI-TOF Analysis of Hemolymph

The Toll pathway was activated in flies using heat-killed *M. luteus*, then incubated at 29°C for 24 h. Hemolymph was extracted as in Lindsay et al. (25), with slight modifications. Hemolymph extracted with glass capillaries from five male flies was pooled and transferred into 0.1% trifluoroacetic acid (TFA)/50% acetonitrile (ACN). One μl of each mixture was spotted on a Bruker MSP 96 ground steel plate, mixed 1:1 with a saturated solution of Universal MALDI matrix (Sigma-Aldrich) in 0.1% TFA/78% ACN, and air-dried. MALDI-TOF spectra were acquired using a Bruker Autoflex mass spectrometer. Data were collected from 1,500 to 10,000 m/z in positive linear mode, and 1,000–5,000 m/z in positive reflectron mode. Peptide calibration standard II (Bruker) was mixed with Universal MALDI matrix and used as an external calibration standard. At least ten independent samples were collected for each genotype. For peptide identification, peaks were matched to those of corresponding peaks in prior studies (13, 25). Representative spectra were visualized using R 3.3.2 and ggplot2 2.2.1 (27, 28).

### Gene Expression Quantitation

The Toll pathway was activated with heat-killed *M. luteus*. Using TRIzol (Ambion), total RNA was extracted 18 h after Toll activation from four to six adult flies (2–5 days old). Next, cDNA was synthesized from 500 ng total RNA using the SuperScript II Reverse Transcriptase kit (Invitrogen). Quantitative RT-PCR was performed on an iQ5 cycler (BioRad) using iQ SYBR Green Supermix (BioRad). Quantification of mRNA levels was calculated relative to levels of the ribosomal protein gene *rp49* using the Pfaffl method (29). Three independent replicates were completed. See Supplemental Table 1 for qPCR primer sequences.

### Hemolymph LC-MS

Flies were challenged with heat-killed *M. luteus* to activate the Toll pathway. Hemolymph was extracted from 100 to 110 each of *w*^1118^, Δ*bbd*, and *Bom*^Δ55*C*^ flies using the same method as in the hemolymph antimicrobial assays, with 50–60 flies processed per Zymo-Spin IC column (Zymo Research) and yielding a total of ~10 μl hemolymph per genotype. Three independent biological replicates were processed for Δ*bbd* and *Bom*^Δ55*C*^, and two independent biological replicates were processed for *w*^1118^. Extracted hemolymph was mixed 1:1 (vol/vol) with denaturing buffer (8 M Urea, 50 mM Tris, pH 7.8, 150 mM NaCl, protease and phosphatase inhibitors) and protein concentration was determined using a BCA assay. For each sample, 40 μg of hemolymph was diluted to 1 M urea using 50 mM ammonium bicarbonate and digested overnight with trypsin (Promega, V511A) at a 1:100 (trypsin:protein) ratio. After digestion, peptides were reduced with 1 mM dithiothreitol at room temperature for 30 min and then alkylated with 5 mM iodoacetamide at room temperature in the dark for 30 min. Formic acid was added to a 0.1% final concentration and peptides were desalted using the C18-Stage-Tip method and then vacuum dried. The dried peptides were reconstituted in 5% formic acid/5% acetonitrile and 1 μg of total peptide for each sample was loaded for MS analysis. Samples were run in technical triplicates on a Q-Exactive mass spectrometer with instrument and chromatography settings as described previously (30), except for the following modifications: the RAW files were analyzed using Andromeda/MaxQuant (version 1.6.7.0) (31) with default settings (32) except the match between the run and LFQ quantitation settings was enabled for label free quantification. Data were searched against a concatenated target-decoy database comprised of forward and reversed sequences from the unreviewed UniprotKB/Swiss-Prot FASTA *Drosophila* database (2019). A mass accuracy of 20 ppm was assigned for the first search and 4.5 ppm for the main search. The statistical analysis was calculated using the DEP analysis R-package (33).

### Bacterial Load Quantification

Bacterial load upon death (BLUD) was obtained as in Duneau et al. (34), with slight modifications. Briefly, flies were infected with *E. faecalis* and vials were monitored every 30 min for newly dead flies. These flies were then individually homogenized with a pestle in 400 μl LB media. Homogenates were also prepared from individual live *w*^1118^ flies 120 h post-infection (hpi). Homogenates were diluted serially in LB and spread on LB agar plates for incubation at 37°C overnight. Colonies were counted manually and the number of viable bacteria per fly was calculated. Data were obtained from three independent experiments.

### Data Analysis

GraphPad Prism 5 was used for statistical tests. Survival data were plotted as Kaplan-Meier curves and were analyzed using the Gehan-Breslow-Wilcoxon test to determine statistical significance. Statistical differences in candidacidal activity were calculated using one-way ANOVA followed by Tukey's test. Multiple Mann-Whitney U tests were used to calculate differences between BLUD samples (*p* = 0.0085 after Šidák correction for multiple comparisons, α = 0.05, *k* = 6). Spearman rank correlation was used to assess the relationship between BLUD and time of death.

## Results

### The *bombardier* Gene Is Specifically Required for Toll-Mediated Defense

The *bombardier* (*bbd*) gene contains a consensus Toll-responsive NF-κB binding site within its promoter region and is strongly expressed upon Toll activation by Gram-positive bacterial infection or other inducers (14, 17, 35, 36). The encoded protein is predicted to be secreted and to generate a mature protein of 222 amino acids with a coiled coil near its C-terminus (37, 38). Orthologs of Bombardier are found across the *Drosophila* genus, but in no other genera (39).

We began our analysis of the *bombardier* gene by generating a null mutant, using CRISPR/Cas9 to delete 1,906 bp encompassing the annotated transcriptional unit. Flies homozygous for this deletion (hereafter Δ*bbd*) were viable and morphologically wild-type. Given that *bombardier* is Toll-inducible, we assayed Δ*bbd* flies for a potential loss-of-function phenotype in Toll-mediated immunity. Specifically, we infected adult Δ*bbd* flies with various pathogens and then monitored survival. Two additional genotypes were used as controls: *w*^1118^ flies, which served as the wild type, and *Bom*^Δ55*C*^ flies, which lack Toll-mediated humoral defenses due to deletion of the 10 of the 12 *Bom* genes (24).

**Figure 1:**
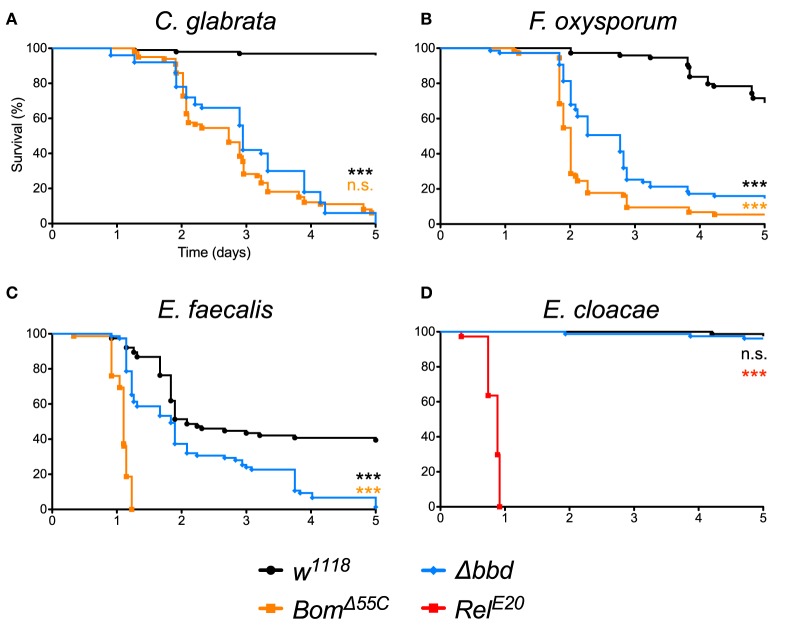
The *bombardier* gene is specifically required for Toll-mediated defense. **(A–D)** Survival curves of flies infected as indicated. The *w*^1118^ strain was the wild-type control; *Bom*^Δ55*C*^ and *Rel*^*E*20^ were the susceptible controls (24, 40). Experiments were completed in triplicate with at least 25 flies per genotype in each replicate. Statistical significance was determined using the Gehan-Breslow-Wilcoxon test and Δ*bbd* is shown relative to *w*^1118^ in black, relative to *Bom*^Δ55*C*^ in orange, and relative to *Rel*^*E*20^ in red (^***^p < 0.0001; n.s., not significant, *p* > 0.05).

As shown in Figure 1, we observed a marked immunodeficiency when Δ*bbd* flies were challenged with representative species for the three classes of microbes against which Toll provides defense. With the yeast *Candida glabrata*, more than 90% of *w*^1118^, but no Δ*bbd* flies, survived 5 days after infection (Figure 1A). In the case of the filamentous fungus *Fusarium oxysporum*, 70% of *w*^1118^ adults, but fewer than 20% of Δ*bbd* adults, were alive 5 days post-infection (Figure 1B). Finally, with *Enterococcus faecalis*, a Gram-positive bacterium, 50% of wild-type flies, but no Δ*bbd* flies, were alive 5 days after infection (Figure 1C).

The impairment of Toll-mediated defenses by deletion of *bombardier* was significant for all three pathogens (*p* < 0.0001). In the case of *C. glabrata*, the immunodeficiency of Δ*bbd* phenocopied that observed for *Bom*^Δ55*C*^ flies (n.s., *p* > 0.05). In contrast, with either *F. oxysporum* or *E. faecalis*, the rate of death was greater for *Bom*^Δ55*C*^ than for Δ*bbd* (^***^p < 0.0001 for both infections). The Δ*bbd* mutant thus displays a substantial, but not complete, loss of Toll-mediated defense.

The expression of *bombardier* is strongly induced by Toll, but not Imd activation (14). We therefore hypothesized that Imd-mediated defenses would not require *bombardier* function. To test this prediction, we infected Δ*bbd* flies with *Enterobacter cloacae*, a Gram-negative bacterium. In this experiment, Δ*bbd* flies were as immunocompetent as *w*^1118^ flies: more than 90% of both genotypes survived at least 5 days post-infection (Figure 1D). In contrast, 100% of *Rel*^*E*20^ flies, which are deficient in Imd signaling (40), succumbed to infection within 1 day. Thus, *bombardier* functions in defense against a range of pathogens for which Toll mediates defense—yeast, filamentous fungi, and Lys-type Gram-positive bacteria—but not against Gram-negative bacteria, against which the Imd pathway is active.

### The Candidacidal Activity of Hemolymph Requires Bombardier, but Neither Drosomycin Nor Metchnikowin

**Figure 2:**
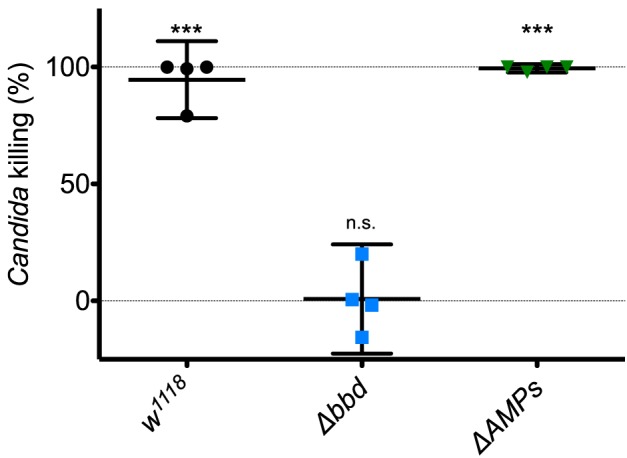
The Toll-induced candidacidal activity of hemolymph requires Bombardier, but neither Drosomycin nor Metchnikowin. Heat-killed *M. luteus* was used to activate the Toll pathway in flies. Hemolymph was extracted from flies 24 h after Toll induction, mixed with *C. glabrata* and incubated for 1 h to allow for killing. The surviving yeast cells were plated, and colonies were counted to determine the level of candidacidal activity in the extracted hemolymph. Colony counts from uninduced *w*^1118^ hemolymph were used as the control for no (0%) killing. Experiments were completed four times, with each point representing one replicate. One-way ANOVA was calculated followed by Tukey's test. Significance is shown relative to the null hypothesis of 0% killing (***p < 0.0001; n.s., not significant, *p* > 0.05). Error bars represent the 95% confidence interval.

Next, we investigated the potential humoral role of Bombardier by preparing and assaying cell-free hemolymph. We have previously shown that hemolymph from wild-type flies exhibits a Toll-dependent and Bomanin-dependent candidacidal activity (25). However, we were also curious as to the identity of the active antifungal component. In particular, we considered the potential role of Metchnikowin (Mtk) and Drosomycin (Drs), two antimicrobial peptides (AMPs) that have documented antifungal activity *in vitro* and are strongly Toll-induced *in vivo* (14, 41, 42). We therefore took advantage of the recently described Δ*AMPs* strain, which is deficient for Mtk and Drs, as well as all other induced AMPs other than the Cecropins (23). Extracting and assaying Toll-induced hemolymph, we found that hemolymph from Δ*AMPs* flies had a killing activity against *C. glabrata* comparable to that of wild-type hemolymph (Figure 2). In contrast, we failed to detect any killing of *C. glabrata* by Δ*bbd* hemolymph. We conclude that Boms and Bombardier, but neither Mtk nor Drs, are required for humoral defense against *C. glabrata*.

### Short-Form Bom Peptides Are Specifically Absent From *Δbbd* Hemolymph

**Figure 3:**
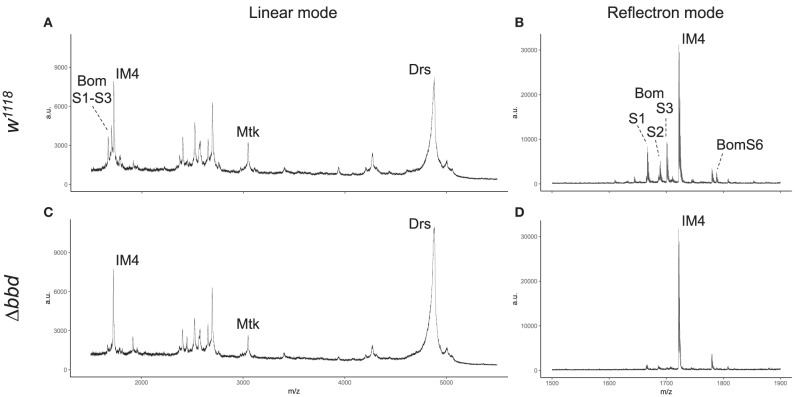
Short-form Bom peptides are specifically absent in hemolymph from Δ*bbd* flies. MALDI-TOF mass spectra of *w*^1118^ **(A,B)** and Δ*bbd* **(C,D)** hemolymph samples were collected in linear **(A,C)** and reflectron mode **(B,D)**. For peptide identification, peaks were matched to those of corresponding peaks in prior studies (13, 25). Spectra were obtained from at least ten independent biological replicates and representative spectra are shown (a.u., arbitrary units; m/z, mass/charge).

MALDI-TOF provides a robust tool for characterizing small (<5,000 MW) peptides present in hemolymph after Toll activation. As shown in Figures 3A,B, such a readout includes the aforementioned AMPs (Mtk and Drs), several short-form Boms (BomS1, S2, S3, and S6; see Supplemental Table 2 for updated Bomanin nomenclature), and other induced peptides (e.g., IM4). We have previously shown that deleting the 55C Bom gene cluster removes the peaks attributable to the short-form Boms, while leaving the remaining signals unaffected (25). Remarkably, analysis of Δ*bbd* hemolymph yielded a similar pattern. As shown in Figures 3C,D, the short-form Boms that were readily detectable in the wild type—S1, S2, S3, and S6—were absent in Δ*bbd* hemolymph, whereas the remaining peptides, including Mtk, Drs, and IM4, displayed a wild-type profile.

Although Δ*bbd* disrupts the accumulation of short-form Bom peptides in hemolymph, this effect does not reflect a disruption in transcription or stability of the corresponding *Bom* mRNAs: robust induction of Toll-regulated genes, including genes of short-form Boms, was readily detectable with qRT-PCR (Supplemental Figure 1).

**Figure 4:**
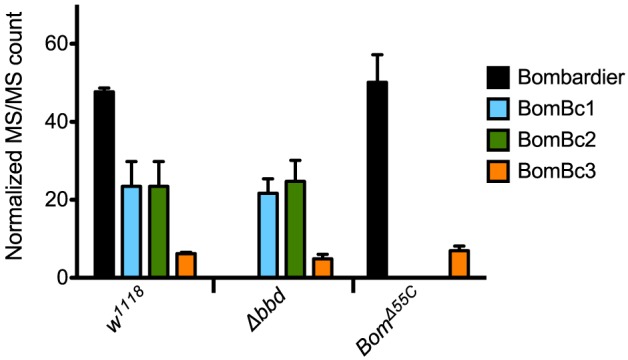
The presence of bicipital Bomanins in hemolymph is unaffected by loss of Bombardier. MS/MS counts for the indicated proteins as determined by Andromeda/MaxQuant were normalized to total MS/MS counts in each run. Error bars represent standard deviation for biological replicates (*n* = 3 for Δ*bbd* and *Bom*^Δ55*C*^, *n* = 2 for *w*^1118^). Supplemental Table 3 shows the full dataset.

Because proteins such as Bombardier and bicipital Boms are too large to be detected by our MALDI-TOF protocol, we used LC-MS to further characterize the relationship between Bombardier and the Boms in hemolymph. For these studies, we prepared Toll-induced hemolymph from three genotypes: *w*^1118^, Δ*bbd*, and *Bom*^Δ55*C*^. In wild-type hemolymph, we readily detected Bombardier protein (Figure 4), consistent with the presence of a canonical secretion signal sequence in the Bombardier coding sequence. Bombardier, like the Boms, is thus secreted into hemolymph upon Toll induction. We also detected all three bicipital Boms—BomBc1, BomBc2, and BomBc3. The LC-MS studies thus complemented the MALDI-TOF studies, with bicipital Boms detected by the former and short-form Boms by the latter (tailed Boms are not detected by either protocol). Next, we assayed Δ*bbd* hemolymph. As expected, Bombardier was not detected. However, the three bicipital Boms were present at comparable levels in wild-type and Δ*bbd* hemolymph (see Figure 4). Combined with the MALDI-TOF studies, these results demonstrate that Δ*bbd* blocks accumulation in hemolymph of short-form, but not bicipital, Boms. Lastly, we analyzed hemolymph from *Bom*^Δ55*C*^ flies, which lack 10 of the 12 *Bom* genes. As expected, the products of the two deleted bicipital genes (*BomBc1* and *BomBc2*) were absent, whereas the product of the remaining bicipital gene (*BomBc3*) was present at wild-type levels (see Figure 4). Turning our attention to Bombardier, we observed no effect of the *55C* Bom deletion. Thus, Bombardier is required for the presence of short-form Boms in hemolymph, but the 55C Boms are not required for the presence of Bombardier.

### Bombardier Mediates Both Infection Resistance and Tolerance

The Δ*bbd* survival phenotype could be due to an inability to control pathogen growth—a defect in resistance—or an inability to endure infection—a defect in tolerance. Because flies lacking Bombardier demonstrate an increased susceptibility to infection and decreased levels of known resistance factors, the short-form Boms, it seemed likely that Δ*bbd* flies, like *Bom*^Δ55*C*^ flies, have a defect in infection resistance. In exploring this hypothesis, we found that the model recently developed by Duneau et al. provided a useful framework (34). Following infection of an individual fly, there are two stereotypic outcomes: either the pathogen replicates, reaches a lethal burden, and the fly dies; or the pathogen is controlled at a level below the lethal burden and the fly survives with a persistent infection. Variation in survival curves for different pathogens and fly genotypes reflects variation in both the time required to reach lethal burden and in the fraction of flies that are able to control the infection before it reaches such a threshold. In cases where a fraction of flies control infection, group survival typically drops after infection and then reaches a plateau (23).

The survival curve for Δ*bbd* flies infected with *E. faecalis* does not plateau (see Figure 1C). Instead, it exhibits a profile that we hypothesize reflects two phases of death. In the first phase, extending roughly 2 days post-infection, some Δ*bbd* flies reach a lethal burden of *E. faecalis* and die, as reflected in a sharp decline in survival; the remainder control the infection. In the second phase, from 2.5 days onward, those flies with a persistent infection die at a reduced but steady rate, due to a defect in tolerance. If this hypothesis is correct, flies dying in the first phase should have a bacterial load upon death (BLUD) comparable to that of wild-type flies dying from infection. Furthermore, those dying in the second phase should have a much lower pathogen burden, comparable to that of wild-type survivors with a persistent infection.

**Figure 5:**
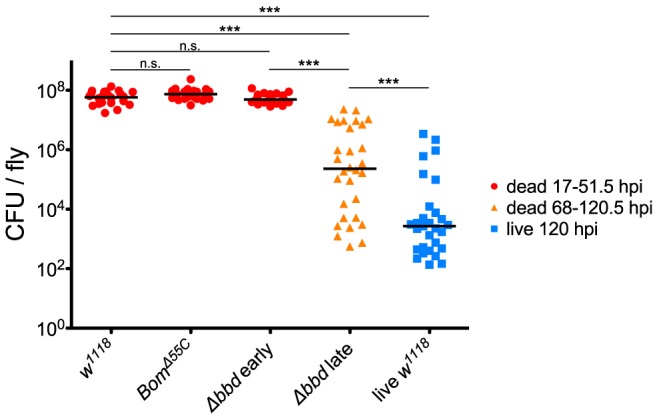
Bombardier mediates both infection resistance and tolerance. Bacterial load upon death (BLUD) of *w*^1118^, *Bom*^Δ55*C*^, and Δ*bbd* flies, plotted by early (17–51.5 hpi, red) or late (68–120.5 hpi, orange) time of death post-infection, as well as bacterial load of live *w*^1118^ flies 120 hpi (blue). Data was obtained and combined from three independent experiments totaling *n* = 26 for *w*^1118^, *n* = 30 for *Bom*^Δ55*C*^, *n* = 33 for Δ*bbd* red, *n* = 30 for Δ*bbd* orange, and *n* = 29 for live *w*^1118^. Black bars indicate median values. Statistics were calculated using multiple Mann-Whitney *U*-tests. For significance, *p* = 0.0085 after Šidák correction for multiple comparisons (α = 0.05, *k* = 6). The pathogen loads of early deaths for *Bom*^Δ55*C*^ and Δ*bbd* were not significantly different from *w*^1118^ (*p* > 0.05). The pathogen load of late Δ*bbd* fly deaths is significantly different from that of the early-death Δ*bbd* and *w*^1118^ groups (***p < 0.0001) and also significantly different from that of live *w*^1118^ flies 120 hpi (***p < 0.0001). Finally, the early-death *w*^1118^ pathogen load was significantly different from that of live *w*^1118^ flies 120 hpi (***p < 0.0001) (hpi, hours post-infection).

To test our predictions regarding pathogen burden, we measured the BLUD of individual flies after infection with live *E. faecalis* and divided the data into two time intervals (Figure 5). For the earlier interval (dead flies obtained between 17 and 51.5 hpi), both *Bom*^Δ55*C*^ and Δ*bbd* bacterial loads upon death were not significantly different from *w*^1118^ (Figure 5, red, *p* > 0.05). For the later time interval (flies obtained between 68 and 120.5 hpi), Δ*bbd* flies perished at significantly lower bacterial loads compared to that of Δ*bbd* flies which died earlier (Figure 5, Δ*bbd* early compared to Δ*bbd* late, *p* < 0.0001), indicating that these two groups die from distinct causes. Importantly, late-death Δ*bbd* flies perished at significantly lower bacterial loads than those of *w*^1118^ suffering early deaths (*p* < 0.0001), demonstrating that Δ*bbd* flies have a defect in tolerance.

Together, the survival curve and BLUD data offer strong support for our two-phase-model: Δ*bbd* flies died early in infection with high bacterial loads, due to a defect in resistance, and died later with lower bacterial loads, reflecting a deficiency in tolerance. However, we note that the bacterial loads of Δ*bbd* flies dying in the later phase were still significantly greater than those of *w*^1118^ flies alive 120 hpi (Figure 5, Δ*bbd* late compared to live *w*^1118^, *p* < 0.0001). This indicates that the later-death Δ*bbd* group has not completely controlled infection compared to the live *w*^1118^ flies, and suggests that both resistance and tolerance contribute to the later Δ*bbd* fly deaths. Although we cannot rule out a minor resurgence in bacterial proliferation preceding late death of *bbd* flies, we note that BLUD and time of death were not significantly correlated for these flies (Supplemental Figure 2, Spearman correlation test, *r* = −0.2654, *p* = 0.1564).

### Immune Activation, Specifically *Bom* Expression, Is Deleterious in the Absence of Bombardier

What is the nature of the tolerance defect we observed in Δ*bbd* flies? More specifically, is their health impaired by an excessive or toxic immune response, or is death due to another class of impaired tolerance (43)? To distinguish between these explanations, we assayed the effect of activating the immune response in Δ*bbd* flies in the absence of infection.

**Figure 6:**
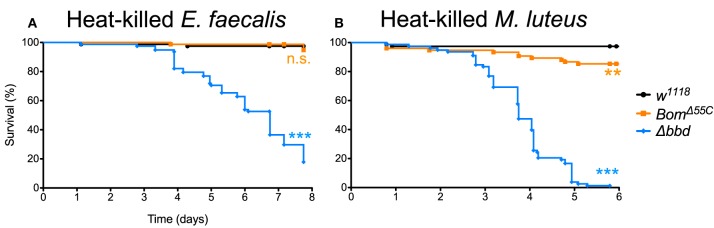
Immune activation is deleterious in absence of Bombardier. Fly survival after introduction of **(A)** heat-killed *E. faecalis* and **(B)** heat-killed *M. luteus*. Experiments were completed in triplicate with at least 25 flies per genotype in each replicate. Statistics were determined using the Gehan-Breslow-Wilcoxon test. Significance is shown relative to *w*^1118^ (***p < 0.0001; n.s., not significant, *p* > 0.05).

When Δ*bbd* flies were challenged with heat-killed *E. faecalis*, we observed a decrease in survival that first was apparent 3 days post-challenge followed by a steady decline in the number of live flies in the following days (Figure 6A), consistent with the timing of the late-phase deaths (see Figure 5). Overall, the death rate was slower than that of live infection, but the extent of killing was similar between heat-killed and live *E. faecalis*: fewer than 20% of flies survived (compare Figures 1C, 6A). In contrast, no effect on survival was observed upon challenge of either *w*^1118^ or *Bom*^Δ55*C*^ flies with heat-killed *E. faecalis*: >95% flies survived seven or more days post-challenge.

The effect of immune stimulation on Δ*bbd* survival was not specific to *E. faecalis*. When we repeated the challenge experiments with heat-killed *Micrococcus luteus*, which activates the Toll response [see Supplemental Figure 1, as well as (25, 44)], the effect on Δ*bbd* survival was again marked: 5 days after challenge, fewer than 5% of Δ*bbd* flies were alive, compared to survival of >95% of *w*^1118^ and 85% of *Bom*^Δ55*C*^ flies over the same period of time (Figure 6B).

**Figure 7:**
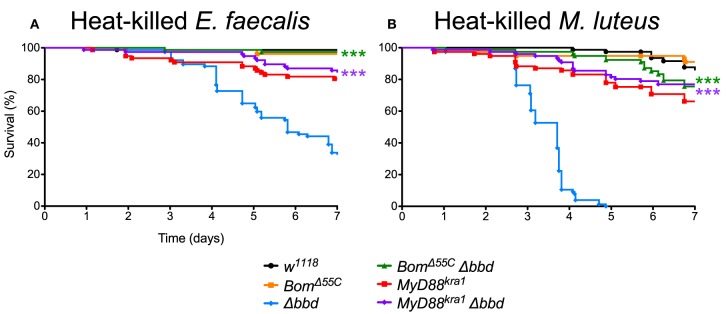
Toll-induced *Bom* expression is responsible for death in immune stimulated Δ*bbd* flies. Survival of flies challenged with **(A)** heat-killed *E. faecalis* and **(B)** heat-killed *M. luteus*. Experiments were completed in triplicate with at least 25 flies per genotype in each replicate. Statistics were determined using the Gehan-Breslow-Wilcoxon test. Significance of double mutant survival curves is shown relative to the survival curve of Δ*bbd* (***p < 0.0001).

As both *M. luteus* and *E. faecalis* induce the Toll pathway, Toll activation could be the key factor in Δ*bbd* mortality. To address this hypothesis, Δ*bbd* flies were crossed with *MyD88*^*kra*1^ (Toll-deficient) flies to generate the *MyD88*^*kra*1^ Δ*bbd* double mutant, and the resulting flies were challenged with heat-killed *E. faecalis* and *M. luteus*. Unlike Δ*bbd* flies, *MyD88*^*kra*1^ Δ*bbd* flies survived challenge with Toll activators (Figures 7A,B). Because blocking the Toll pathway with *MyD88*^*kra*1^ rescues the Δ*bbd* phenotype triggered by heat-killed bacteria (*p* < 0.0001 compared to Δ*bbd, p* > 0.05 compared to *MyD88*^*kra*1^ for both heat-killed bacteria), we conclude that Toll activation underlies the death of Δ*bbd* flies in the absence of infection.

As described above, *Bom* genes are transcribed in Δ*bbd* flies (Supplemental Figure 1), but short-form Bom peptides do not appear in hemolymph (Figure 3). This suggests a mislocalization of these peptides, perhaps in an unprocessed or misfolded state. Given that short-form *Bom* genes are among the most abundantly transcribed genes after infection (17, 36), such mislocalized or misfolded Boms could rapidly accumulate to high levels in Δ*bbd* flies. Could this explain the death of Δ*bbd* flies upon immune stimulation? To address this question, we generated *Bom*^Δ55*C*^ Δ*bbd* double mutants and assayed the effect of immune induction alongside both *Bom*^Δ55*C*^ and Δ*bbd* flies (Figures 7A,B). The result was unequivocal: introducing *Bom*^Δ55*C*^, which deletes all of the short-form Boms, eliminated the effect of Δ*bbd* on survival following immune stimulation (*p* < 0.0001 compared to Δ*bbd, p* > 0.05 compared to *Bom*^Δ55*C*^ for both heat-killed bacteria). The fact that *Bom*^Δ55*C*^ is epistatic to Δ*bbd* demonstrates that Toll-driven expression of *Bom* genes is specifically responsible for the death of immune-stimulated Δ*bbd* flies.

## Discussion

The results presented in this study identify a key factor that regulates humoral and Bom-mediated defense in *Drosophila*. We demonstrate that Δ*bbd* flies are defective in resistance to pathogens controlled by the Toll pathway. The results support the hypothesis that this defect results from the absence of short-form Boms in Δ*bbd* hemolymph. Absence of Boms is sufficient to cause a defect in resistance (24) and Δ*bbd* hemolymph appears to be lacking the short-form Boms but no other component, save Bombardier itself. Furthermore, Δ*bbd* phenocopies *Bom*^Δ55*C*^ with regard to survival after *C. glabrata* infection, and resistance to *C. glabrata* can be restored in *Bom*^Δ55*C*^ flies by expression of short-form Boms (25). Finally, Δ*bbd* hemolymph lacks candidacidal activity, which is dependent on short-form Bom peptides (25) and which we show here does not require Drs or Mtk.

For pathogens other than *C. glabrata*, the effect of deleting Bombardier is less severe than that of deleting the ten *Bom* genes clustered at 55C. Our mass spectrometry data suggest an explanation. Whereas, short-form Boms are absent from Δ*bbd* hemolymph, bicipital Boms are present. (Tailed Boms were not detected with either mass spectrometry method.) Therefore, we postulate that the bicipital Boms, which are not required for resistance to *C. glabrata* (25), are functional against other pathogens. This would explain why Δ*bbd* flies are more resistant than *Bom*^Δ55*C*^ flies upon infection with *E. faecalis* or *F. oxysporum* (Figure 1). In this regard, we note that Bombardier and all three forms of Bom proteins—short, tailed, and bicipital—are found across the *Drosophila* genus, supporting the notion that all three classes of Boms are immunoprotective and therefore maintained across the *Drosophila* genus.

It might appear that our discovery of Bombardier was serendipitous, given our role in defining the *Bomanin* gene family (24, 25). In hindsight, however, the link was forged in our approach. We selected *CG18067* from the most strongly inducible Toll-regulated loci, a group that also includes eight of the *Bomanin* genes. Next, we engineered a CRISPR/Cas9 deletion of *CG18067* and assayed this knockout with the identical set of pathogens that we had used for the *Bom*^Δ55*C*^ deletion, screening for loss of survival upon infection. Having examined a gene that is as strongly induced as the Bomanins, present in the same range of species as the Bomanins, and with a spectrum of loss-of-function phenotypes similar to that of the Bomanins, it is not particularly surprising that we would find ourselves studying a gene that affects the Bomanins.

### Bombardier Function and Structure

What is the function of Bombardier? Deleting the gene results in the absence of short-form Boms from hemolymph, an effect we find is at the level of protein. Other mature immune peptides are present at normal levels in the hemolymph, and there is thus no general defect in translation, secretion, or processing. Based on these findings, we propose that Bombardier normally functions either to chaperone short Boms as they are secreted from the fat body into the hemolymph or, alternatively, to protect the Boms from misfolding or aggregation while in the hemolymph. We further hypothesize that it is the ectopic localization or aberrant form of short-form Boms in Δ*bbd* flies that generates morbidity upon Toll pathway activation. In support of this idea, we showed that *Bom* expression underlies the lethality observed in Δ*bbd* flies (Figure 7). Whether the short-form Boms physically interact with Bombardier, perhaps in the context of a larger antimicrobial complex, is currently unknown.

Activation of Toll-like receptor (TLR) signaling is important for innate immunity, but induction of the pathway can lead to autoimmune disorders and chronic inflammatory disease (45–48). Here we report an autoimmune activity driven by Toll-induced *Bom* expression in flies lacking a downstream pathway component, Bombardier. To what extent this parallel can be exploited in the context of understanding autoimmune disorders promises to be a significant focus for future investigation.

## Data Availability Statement

The LC-MS datasets generated for this study can be accessed on the MassIVE data repository using the accession identifier: MSV000084509.

## Author Contributions

SL and SW: conceptualization, writing—original draft, and visualization. SL, AF, and EB: methodology. SL and AF: formal analysis. SL, AF, and LC: investigation. SL, AF, LC, EB, and SW: writing—review and editing. EB and SW: supervision and funding acquisition.

### Conflict of Interest

The authors declare that the research was conducted in the absence of any commercial or financial relationships that could be construed as a potential conflict of interest.

## Footnotes

^1^http://blogs.cornell.edu/drosophila/dssc-cornmeal-recipe/
