# Supplementary material for: Bombardier Enables Delivery of Short-Form Bomanins in the Drosophila Toll Response
Source: Front Immunol. 2020 Jan 10;10:3040. doi: 10.3389/fimmu.2019.03040 (PMC6965162; doi:10.3389/fimmu.2019.03040)
Supplement: Supplementary file 4 [file Data_Sheet_1.PDF]

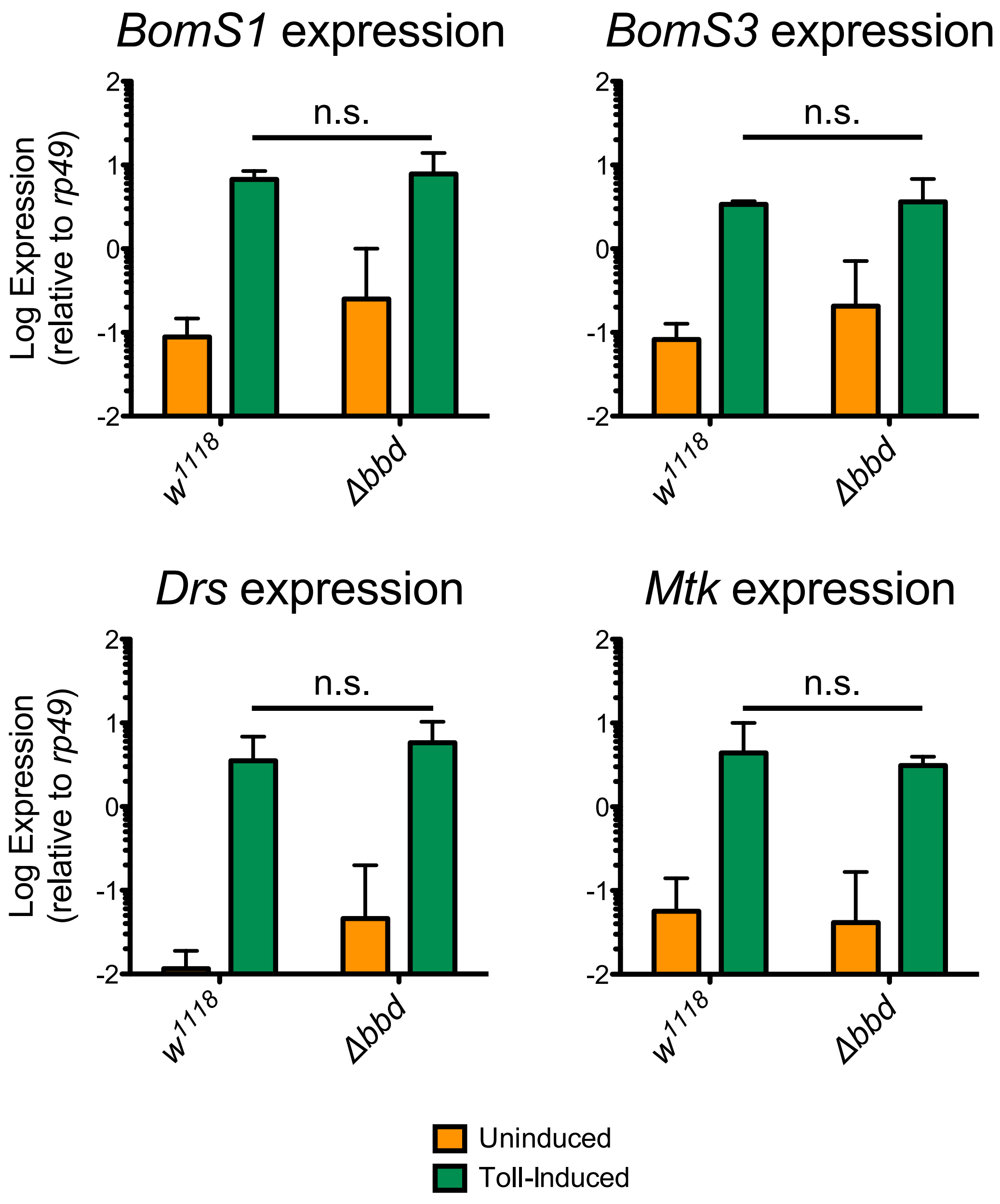

Supplemental Figure 1. Δ*bbd* deletion does not affect Toll signaling.
qRT-PCR analysis of uninjured flies and flies 18 hours after challenge with heat-killed *M. luteus*. Gene mRNA levels were calculated relative to levels of *rp49*, a ribosomal gene. Experiments were completed with independent biological triplicates. Two-way ANOVA was calculated followed by Tukey's test, indicating that Toll-induction is a significant variable for differences in mRNA levels, but fly genotype is not a significant variable. Error bars show 95% confidence interval. (n.s. = not significant, $p>0.05$)
