# Supplementary material for: Bombardier Enables Delivery of Short-Form Bomanins in the Drosophila Toll Response
Source: Front Immunol. 2020 Jan 10;10:3040. doi: 10.3389/fimmu.2019.03040 (PMC6965162; doi:10.3389/fimmu.2019.03040)
Supplement: Supplementary file 5 [file Data_Sheet_2.PDF]

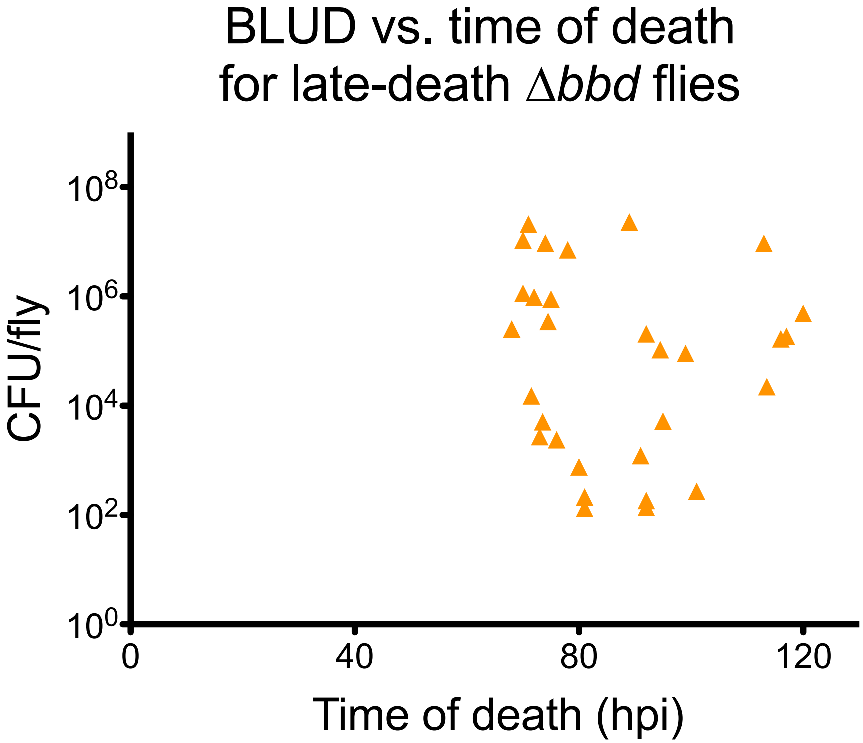

Supplemental Figure 2. Lack of correlation between BLUD and time of death of late-death Δ*bbd* flies. BLUD counts of late-death Δ*bbd* flies (see Figure 5) were plotted with time of death. The time of death and BLUD were not significantly correlated (Spearman correlation test, r = -0.2654, p = 0.1564)
